# Ginkgolide A enhances cardiomyocyte differentiation from pluripotent stem cells by targeting cytochrome c to attenuate intrinsic apoptosis

**DOI:** 10.1016/j.jbc.2026.113224

**Published:** 2026-06-06

**Authors:** Wenjie Xu, Xiaolong Wu, Zuo Lei, Xiaoyu Dang, Hongzhao Shi, Jiannan Li, Liming Yuan, Yaya Zhao, Wenhao Li, Jing Chen, Na Li, Jinlian Hua

**Affiliations:** 1 College of Veterinary Medicine, Shaanxi Centre of Stem Cells Engineering and Technology, Northwest A&F University, Yangling, Shaanxi, China; 2 Shaanxi Stem Cell Application Engineering Research Center, Shaanxi Jiuzhou Biomedical Science and Technology Group, Xi'an, Shaanxi, China

**Keywords:** pluripotent stem cells, ginkgolide A, cardiomyocytes, apoptosis, natural product, cell manufacturing

## Abstract

Efficient differentiation of pluripotent stem cells (PSCs) into functional cells is critical for regenerative medicine and biomanufacturing, yet is often hampered by apoptosis. Ginkgolide A (GA) is a diterpene lactone derived from *Ginkgo biloba* leaves and a member of the ginkgolide family, compounds known for diverse biological activities including neuroprotection and cardiovascular regulation. However, the mechanism by which ginkgolides influence the directed differentiation of stem cells remains unclear. Here, using a CRISPR-Cas9-engineered TNNT2-mCherry reporter PSC line and a defined cardiac differentiation protocol, we screened ginkgolides for their effects on cardiomyocyte (CM) production. Results demonstrated that GA significantly enhanced CM induction efficiency and accelerated the onset of spontaneous beating. Concurrently, GA effectively inhibited apoptosis during differentiation and RNA-Seq results also revealed that GA orchestrates stage-specific upregulation of antiapoptotic genes (*e.g.*, *MCL1* and *XIAP*) and core cardiogenic transcription factors (*NKX2-5* and *GATA4*). Molecular docking predictions suggested a high binding potential between GA and cytochrome c, suggesting GA might inhibit the intrinsic mitochondrial apoptosis cascade by interfering with cytochrome c's binding to apoptotic peptidase activating factor-1 (APAF-1). These data demonstrate that GA enhances the differentiation of PSCs into CMs, potentially through its antiapoptotic effect. This mechanism highlights its potential as a safe culture additive to boost cell survival and yield for large-scale biomanufacturing and tissue engineering applications.

Ischemic heart disease, particularly myocardial infarction, remains a leading cause of mortality worldwide, primarily due to the irreversible loss of cardiomyocytes (CMs) (1). Given the limited regenerative capacity of adult CMs, human pluripotent stem cell (hPSC)-derived CMs have emerged as a promising cell source to replenish the damaged myocardium (2, 3, 4). Although *in vitro* differentiation protocols have achieved high efficiency in experimental settings, two intertwined challenges, low differentiation efficiency, and massive cell death during the differentiation process, remain when it comes to clinical translation and large-scale manufacturing of these cells (5, 6). Among these, apoptosis—a programmed cell death mechanism—is frequently triggered during the delicate transition from pluripotency to a committed cardiac lineage, leading to substantial loss of the final cell product and compromising therapeutic efficacy (7, 8).

*Ginkgo biloba*, a “living fossil” with a long history of culinary and medicinal use in Asia, provides abundant sources of bioactive compounds for the clinical and agricultural use recently (9). While crude extracts of the leaves are used traditionally, its purified diterpene lactones, including Ginkgolide A (GA), are of particular interest for diverse pharmacological activities (10). Notably, GA exhibits potent cardioprotective effects in attenuating pathological myocardial remodeling and heart failure (11). At the cellular level, GA has been shown to inhibit apoptosis in neurons and endothelial cells *via* modulation of the PI3K/Akt pathway and reduction of endoplasmic reticulum stress (12, 13, 14). These findings suggest that GA may possess a general antiapoptotic capability. As a natural phytochemical component, GA offers the advantage of being more readily accepted by regulatory frameworks for cell manufacturing (15). Despite previous progress, the direct effect of GA on pluripotent stem cell (PSC)-derived CMs and its underlying molecular mechanism remain largely unexplored.

Here, we hypothesized that GA, with its established heart-protective and likely antiapoptotic properties, could enhance the production of CMs from PSCs. To test this, we employed an integrated strategy: the use of a CRISPR-Cas9-generated CM reporter stem cell line for high-throughput phenotypic screening. We then performed transcriptomic profiling (RNA-Seq) across key developmental stages to map the global impact of GA, followed by informatics-based target prediction and experimental validation. Our findings demonstrate that GA significantly promotes CM differentiation efficiency by attenuating apoptosis. Mechanistically, we provide evidence that GA exerts its effects by directly targeting cytochrome c (CYC), thereby inhibiting the intrinsic mitochondrial apoptosis pathway (16). This study unveils a novel role for GA in regulating cell fate decisions and positions this plant-derived terpenoid as a promising candidate culture additive. It offers a tangible strategy to improve cell yield in biomanufacturing applications, bridging the gap between traditional herbal medicine and modern cellular agriculture technology.

## Results

### CMs derived from human pluripotent stem cells

**Figure 1:**
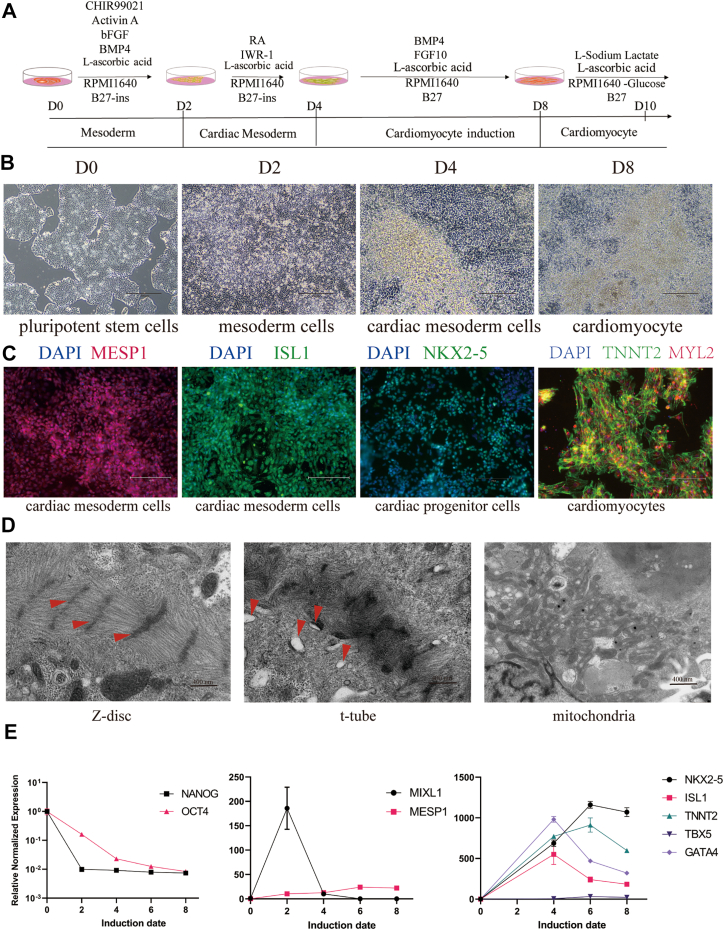
**Direct differentiation and characterization of human pluripotent stem cell (hPSC)-derived cardiomyocytes.***A*, schematic of the modified differentiation protocol. *B*, phase-contrast images showing cellular morphology at key induction time points. The scale bar represents 300 μm. *C*, immunofluorescence analysis of stage-specific cell markers. The scale bar represents 100 μm. *D*, transmission electron microscopy of the specific subcellular structure of hPSC-derived cardiomyocytes. The *red triangle* targets the specific structure labeled under the figure. The scale bar represents 400 nm. *E*, qRT-PCR results of marker genes at different induction time points of stem cell, mesoderm, and cardiomyocytes. The induction date is listed at the x axis. Data indicate mean ± SD, n = 3. qRT-PCR, quantitative reverse transcription polymerase chain reaction.

Based on previous studies (17, 18, 19), we used a chemically defined differentiation protocol for the PSCs used in this study (Fig. 1*A*). In this protocol, hPSCs are directed to differentiate through stages including mesoderm, cardiac mesoderm, and cardiac progenitor cells (CPCs) into CMs *via* small molecule inhibitors and cytokines (Fig. 1*A*). Upon activation of the Wnt pathway with CHIR99021 (the GSK3 inhibitor) and cytokines bone morphogenetic protein 4 (BMP4), Activin A, and fibroblast growth factor-basic, hPSCs differentiated into mesoderm cells. As stem cell colonies disappear, rhomboid-shaped cells emerge (Fig. 1*B*). The quantitative reverse transcription polymerase chain reaction (qRT-PCR) results confirmed high expression of primitive streak marker *MIXL1* and mesoderm markers *MESP1* and *TBXT* on induction Day 2 (Fig. 1*E*). Subsequently, Wnt pathway inhibition with IWR-1 and supplementation with retinoic acid promoted cardiac mesoderm formation (20). By Day 4, cells appeared tightly packed and multilayered (Fig. 1*B*). Cells at this stage expressed high levels of cardiac mesoderm markers ISL1 and MESP1 (Fig. 1*C*).

From Day 4, fibroblast growth factor 10 and BMP4 were added to promote differentiation of cardiac mesoderm into CPCs (21, 22). Studies have shown that during this transition, a mixed population of cardiac mesoderm, CPCs, and early CMs exists, with CPCs undergoing both proliferation and differentiation (23, 24, 25). On Day 6, cells highly expressed CPC markers *NKX2-5*, *ISL1*, *GATA4*, and *TBX5*, as well as cardiac mesoderm marker *MESP1* and early CM marker *TNNT2* (Fig. 1*E*).

By Day 8, the differentiation from cardiac mesoderm to CPCs was predominantly complete. Cells exhibited a spindle-like morphology and displayed stable, rhythmic spontaneous contractions. The qRT-PCR results confirmed high expression of the CM marker *TNNT2* (Fig. 1*E*). Immunofluorescence staining for TNNT2 protein revealed distinct myofibrillar structures (Fig. 1*C*). Following 2 days of sodium lactate selection, the CMs were kept in maintenance medium, and cell maturity increased significantly by Day 14. Transmission electron microscopy showed aggregated and fused mitochondria, formation of distinct Z-disc sarcomeric structures, and the emergence of T-tubules characteristic of maturing CMs (Fig. 1*D*).

### TNNT2 reporter cell line

**Figure 2:**
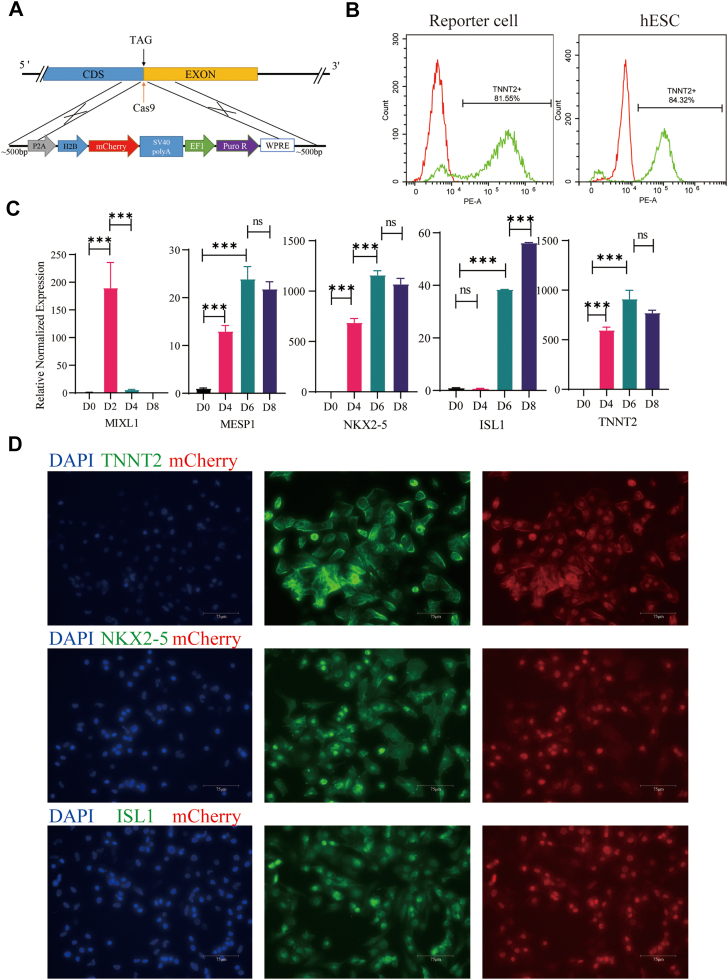
**Characterization of TNNT2 reporter cell line-derived cardiomyocytes.***A*, schematic of reporter gene knock in *via* CRISPR-Cas9. *B*, flow cytometric analysis comparing the cardiomyocyte induction efficiency between the reporter cell line and the unmodified stem cell line. The PE-A track represents the mCherry fluorescence on the Reporter cell, and the Alexa Fluor 568 fluorescence on the hESC. *C*, stage-specific qRT-PCR analysis of mesoderm, cardiac mesoderm, and cardiomyocytes. Data are presented as mean ± SD, n = 3. The *p* values were calculated by using one-way ANOVA analysis followed by Tukey’s *post hoc* test for multiple comparisons. Significance levels: ∗*p* < 0.05; ∗∗*p* < 0.01;∗∗∗*p* < 0.001. *D*, immunofluorescence staining of cardiomyocytes markers in the reporter cell line-derived cardiomyocytes. The scale bar represents 75 μm. hESC, human embryonic stem cell; qRT-PCR, quantitative reverse transcription polymerase chain reaction.

Significant upregulation of *TNNT2* gene expression is a key indicator of successful CM differentiation (26). Using CRISPR-Cas9 gene editing, a red fluorescent reporter gene (*H2B-mCherry-PuroR*) was introduced just before the stop codon of the *TNNT2* ORF in hPSCs (Figs. 2*A* and S1, *A* and *B*). Flow cytometry analysis on Day 10 showed that the CM induction efficiency from mCherry-labeled hPSCs was comparable to that of unedited human embryonic stem cells (hESCs), both exceeding 80% (Fig. 2*B*).

The qRT-PCR results for the reporter line showed high expression of *MIXL1* on Day 2 and *MESP1* on Day 4 at the mesoderm stage, high expression of *ISL1* and *NKX2*-5 at the progenitor stage on Day 6, and *TNNT2* mRNA expression initiating during the cardiac mesoderm stage on Day 4 (Fig. 2*C*). Immunofluorescence staining confirmed colocalization of the red fluorescent signal (nuclear, due to H2B tag) with CM markers TNNT2, NKX2-5, and ISL1 in differentiated cells (Fig. 2*D*).

Collectively, these results demonstrate the successful generation of a PSC-derived TNNT2 fluorescent reporter line, providing a robust tool for tracking CM differentiation in real time.

### Screening ginkgo chemical components for effects on CM induction using the reporter line

**Figure 3:**
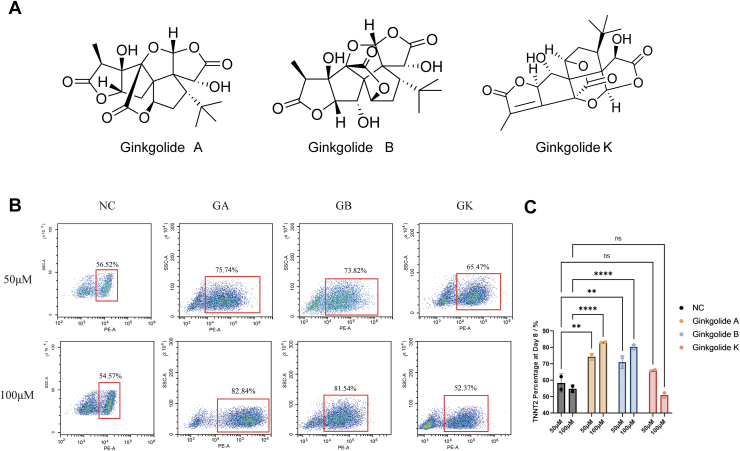
**The effects of ginkgolides on cardiomyocyte differentiation.***A*, representative structures of the three main diterpene lactones in the ginkgolide family. *B* and *C*, effects of the three major ginkgolide components on cardiomyocyte induction efficiency at the indicated concentrations of 50 μM and 100 μM. The PE-A track represents the mCherry fluorescence on the Reporter cell. Data are presented as mean ± SD, n = 3. The *p* values were calculated by using One-way ANOVA analysis followed by Tukey’s *post hoc* test for multiple comparisons. Significance levels: ∗*p* < 0.05; ∗∗*p* < 0.01;∗∗∗*p* < 0.001.

Previous studies indicate that GA, GB, and GK exhibit neuroprotective effects and attenuate apoptosis (12, 27, 28), with GA and GK also showing cardioprotective properties (29, 30). GA, GB, and GK share a typical cage-like diterpene core skeleton with multiple lactone rings but differ in hydroxyl group number and saturation (Fig. 3*A*). GA has one hydroxyl, GB has two, while GK has two hydroxyls and an additional carbon-carbon double bond, existing as an α, β-unsaturated lactone.

**Figure 4:**
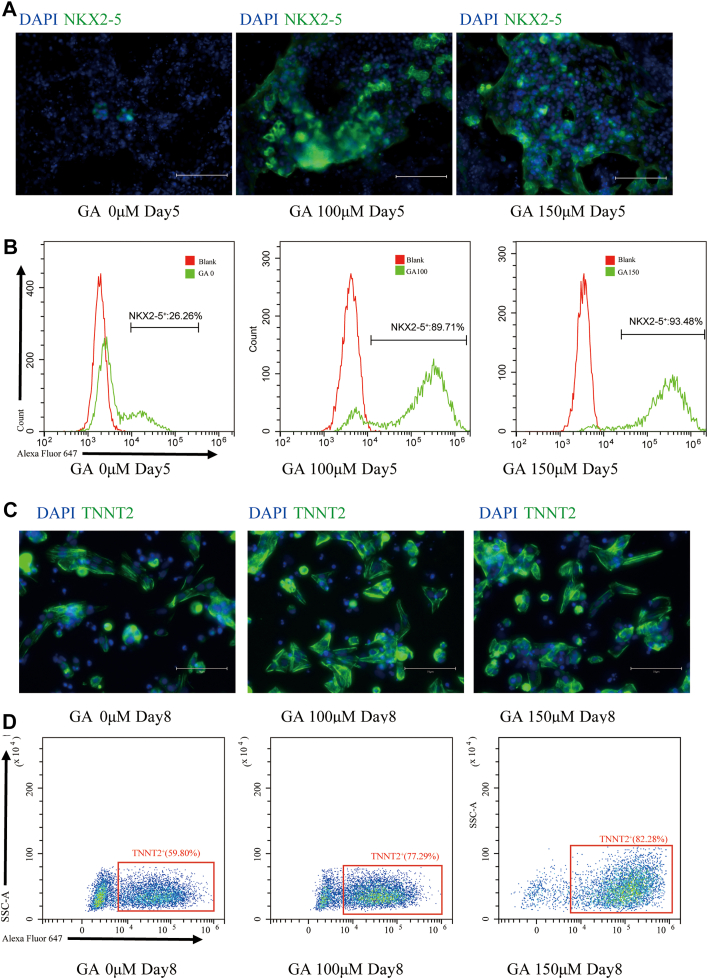
**Ginkgolide A modulates cardiac protein expression and differentiation efficiency.***A*, NKX2-5 immunofluorescence staining across indicated ginkgolide A concentrations. The scale bar represents 75 μm. *B*, induction efficiency analysis by flow cytometry on day 5, based on NKX2-5^+^ cells. *C*, TNNT2 immunofluorescence staining across indicated ginkgolide A concentrations. The scale bar represents 75 μm. *D*, induction efficiency analysis by flow cytometry on day 8, based on TNNT2^+^ cells.

To investigate which ginkgolide influences CM differentiation, we used the reporter system to assess the effects of these three ginkgolides on differentiation efficiency. The cells just before selection was set as the comparative time point. Results showed that GA and GB significantly enhanced CM induction efficiency, while GK's effect was not significant (Fig. 3, *B* and *C*). Notably, GA addition led to a unique change: the first occurrence of spontaneous beating was observed on Day 5 (Fig. S1*C*, Videos S1, and S2), specifically at concentrations of 100 μM and 150 μM. Analysis of the key cardiac transcription factor NKX2-5 (31) showed that GA significantly increased its expression (Fig. 4, *A* and *B*). Consistent with this, immunofluorescence staining and flow cytometry for TNNT2 also showed significant enhancement.

In summary, screening of ginkgolide family members with the reporter line identified GA as an effective supplement for promoting CM differentiation.

### GA inhibits apoptosis during stem cell differentiation

**Figure 5:**
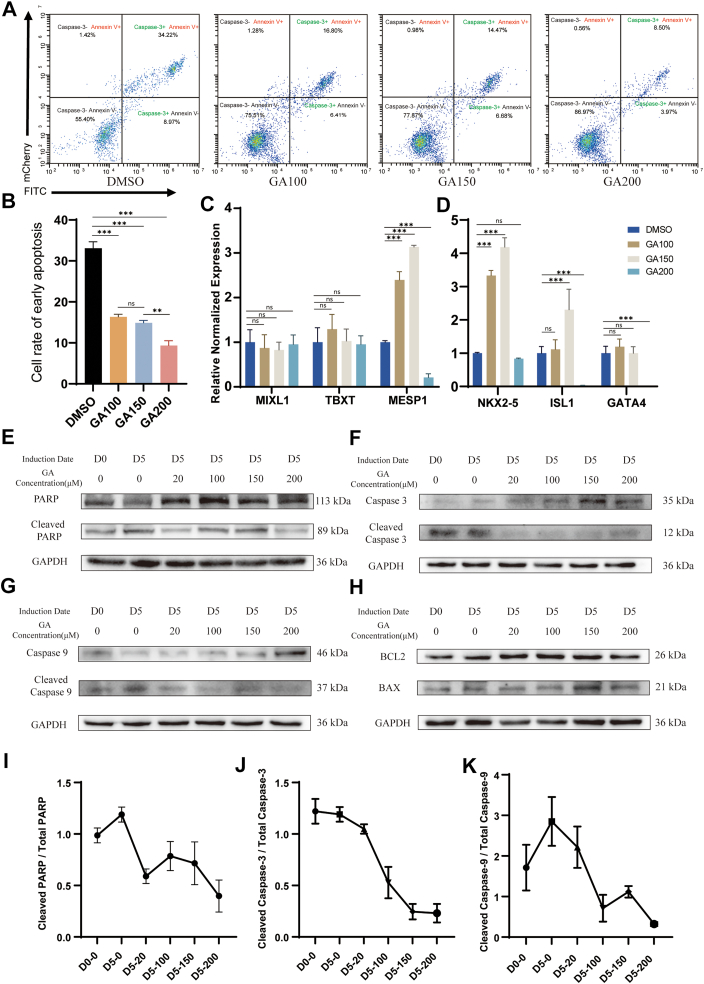
**Ginkgolide A attenuates apoptosis and modulates differentiation gene programs.***A* and *B*, apoptosis assessment by flow cytometry (Annexin V/Caspase 3). Annexin V was labeled with mCherry, and the GreenNuc Caspase-3 was activated upon cleavage by Caspase-3, after which GreenNuc binds to DNA and produces *bright green fluorescence* on the FITC channel. Data are presented as mean ± SD, n = 3. The *p* values were calculated by using one-way ANOVA analysis followed by Tukey’s *post hoc* test for multiple comparisons. Significance levels: ∗*p* < 0.05; ∗∗*p* < 0.01;∗∗∗*p* < 0.001. *C* and *D*, qRT-PCR analysis of marker gene expression for mesoderm (*C*) and cardiac progenitor cells (*D*) under ginkgolide A treatment. Data are presented as mean ± SD, n = 3. The *p* values were calculated by using one-way ANOVA analysis followed by Tukey’s *post hoc* test for multiple comparisons. Significance levels: ∗*p* < 0.05; ∗∗*p* < 0.01; ∗∗∗*p* < 0.001. *E*–*H*, Western blot analysis of key apoptosis-related proteins at different differentiation stages (day 0, D0 and day 5, D5) and ginkgolide A concentrations (GA, 0, 20, 100, 150, and 200 μM). Target proteins include total and cleaved PARP (*E*), total and cleaved Caspase-3 (*F*), total and cleaved Caspase-9 (*G*), antiapoptotic protein BCL2 and proapoptotic protein BAX (*H*). GAPDH was used as the internal loading control to confirm equal protein loading. *I*–*K*, densitometric quantification was performed based on the Western blot bands in (*E*–*G*). The graphs display the relative ratio of cleaved protein to its corresponding total protein, including cleaved PARP/total PARP (*I*), cleaved Caspase-3/total Caspase-3 (*J*), and cleaved Caspase-9/total Caspase-9 (*K*), to reflect the activation level of the core apoptosis executive pathway. Data are expressed as mean ± SD from three independent experiments. PARP, poly(ADP-ribose) polymerase; BAX, BCL2-associated X protein.

To investigate the effect of GA on apoptosis, we collected cells on Day 5 from negative control (dimethyl sulfoxide), 100 μM GA, and 150 μM GA groups and assessed Caspase-3 activity and Annexin V fluorescence by flow cytometry. Flow cytometry results showed that Annexin V^+^/Caspase-3^+^ double-positive cells, which exhibit phosphatidylserine externalization and activated Caspase-3, were defined as apoptotic cells (16, 32). In the negative control group, the proportion of apoptotic cells on Day 5 was 34.2%, which significantly decreased to 16.8% and 8.5% with 100 μM and 150 μM GA treatment, respectively (Fig. 5, *A* and *B*). Concurrently, expression of the cardiac mesoderm marker *MESP1* on Day 4 (Fig. 5*C*) and CPC markers *NKX2-5* and *ISL1* on Day 5 (Fig. 5*D*) was significantly increased, indicating enhanced induction efficiency for these stages. Notably, GA treatment did not significantly affect mesoderm marker genes *MIXL1* and *TBXT*, but high concentrations (200 μM) of GA significantly impeded the expression of cardiac mesoderm marker *MESP1* on Day 4 and key CPC genes *ISL1* and *GATA4* on Day 5. These results indicate that while GA's antiapoptotic effect is beneficial at optimal doses, a basal level of developmental apoptosis may be physiologically necessary for proper lineage commitment, and its complete abrogation could be detrimental.

We further validated GA's effect on the apoptosis signaling pathway by Western blotting (Fig. 5, *E*–*H*). During differentiation of day 5 (D5), compared to undifferentiated PSCs (D0), levels of Cleaved PARP, Cleaved Caspase-3, and Cleaved Caspase-9 proteins were markedly increased. Densitometric analysis further confirmed a significant, dose-dependent decrease in the ratio of cleaved to total PARP, Caspase-9 and Caspase-3 upon GA treatment (Fig. 5, *I*–*K*). GA supplementation significantly reduced the levels of these cleaved apoptotic proteins in differentiating cells. Furthermore, the reduction in cleaved proteins showed a dose-dependent response to GA concentration. However, BCL2 and BAX protein levels showed no significant change with GA treatment.

In summary, GA inhibits apoptosis by attenuating Caspase-3 cleavage and promotes the generation of cardiac mesoderm and CPCs.

### RNA-Seq analysis of GA-treated PSC-derived CMs at different stages

**Figure 6:**
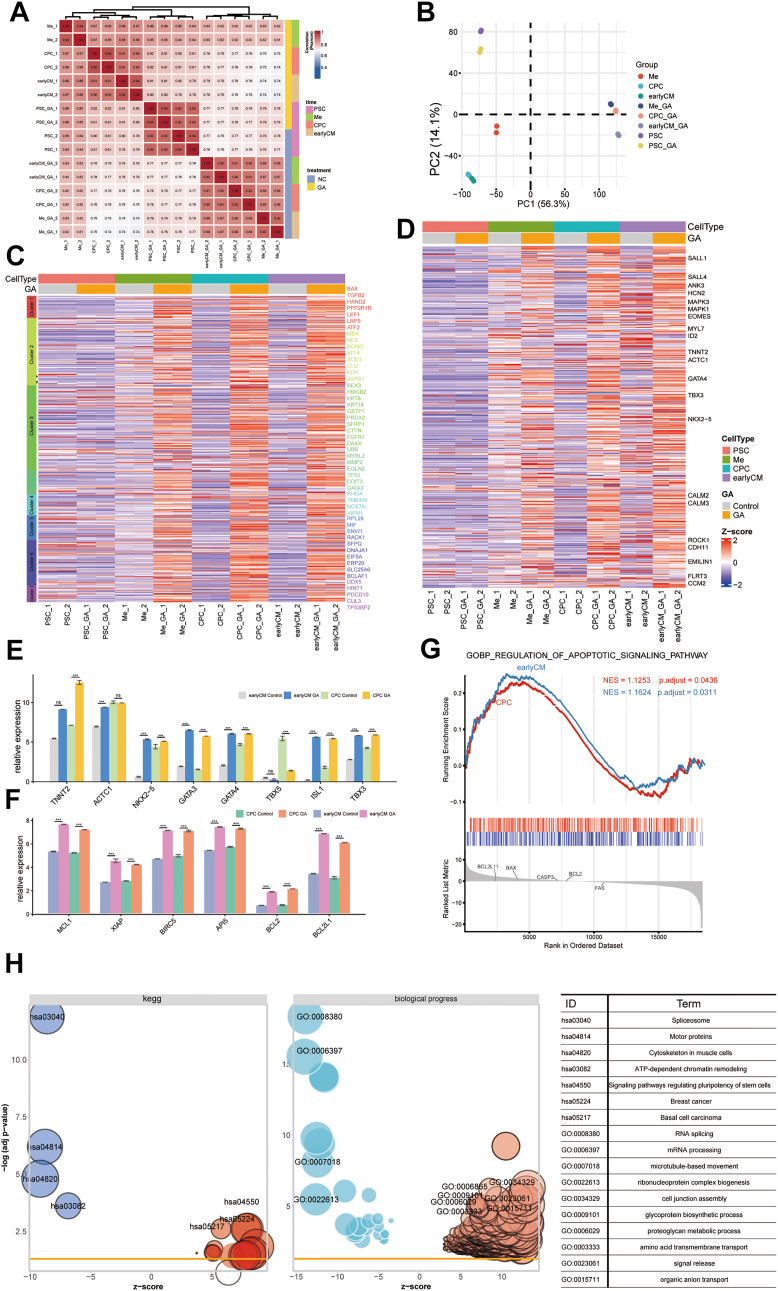
**RNA-Seq analysis of the effect of GA on cardiomyocyte differentiation from PSCs.***A*, Pearson correlation coefficient matrix for PSCs, cardiac mesoderm (Me), cardiac progenitor (CPC), and early cardiomyocytes (early CM), with or without GA treatment (suffix “_GA”). Color intensity indicated the correlation coefficient, with *red* representing higher correlation and *blue* representing lower correlation. *B*, PCA of RNA-Seq samples across all stages of Me, CPC, and early CM with or without GA treatment. *C*, heatmap showing key differences across Me, CPC, and early CM with or without GA treatment. Representative genes of each cluster were listed on the *left*. Z-score values ranging from −2 to 2 indicate scaled expression levels. *D*, heatmap of genes associated with cardiomyocytes differentiation or heart development-related GO terms. *E*, relative expression levels of core cardiomyocytes development genes in PSC-derived CMs across the three stages. *F*, relative expression of genes involved in negative regulation of apoptosis in PSC-derived CMs across the three stages. *G*, GSEA results for the three stages. The cardiac mesoderm stage (Me) was not included as *p* > 0.05. *Red* and *blue lines* represent CPC and earlyCM, respectively. *H*, KEGG and GO biological process enrichment analysis of CPC with GA supplement. Positive or negative Z scores indicate activation or suppression of the corresponding term. Term ID and symbol were listed on the table. GA, ginkgolide A; PSC, pluripotent stem cell; PCA, principal component analysis; GO, Gene Ontology; CM, cardiomyocyte; GSEA, gene set enrichment analysis; KEGG, Kyoto Encyclopedia of Genes and Genomes.

To further investigate the role of GA in CM differentiation and development, we collected samples at multiple developmental time points, including the cardiac mesoderm, CPCs, and early CMs to performed bulk RNA-Seq. Pearson correlation analysis (Fig. 6*A*) and principal component analysis (PCA) revealed that GA treatment had a minimal impact on the transcriptome of PSCs, as evidenced by the close clustering of samples in the PCA plot (Fig. 6*B*). In contrast, at the stages of cardiac mesoderm, CPCs, and early CMs, GA treatment significantly altered the transcriptional expression profiles, indicating that the effects of GA are developmentally stage-dependent.

Subsequently, using GA supplementation at different time points as experimental conditions, we conducted differential gene expression analysis followed by Gene Ontology (GO) enrichment analysis (Fig. 6*C*). A total of seven clusters were identified, including “apoptotic process involved in development”, “apoptotic process involved in morphogenesis”, “negative regulation of neuron apoptotic process”, “extrinsic apoptotic signaling pathway”, “regulation of neuron apoptotic process”, “neuron apoptotic process”, and “regulation of apoptotic signaling pathway”. Of note, multiple clusters were associated with apoptotic processes, which may be attributed to the conserved functions of apoptosis-related regulatory genes in CMs. The presence of terms related to “neuron apoptotic process” likely reflects the inherent functional conservation of apoptosis-regulatory genes across different cell types and the overlapping nature of GO annotations, rather than a specific neuronal differentiation effect.

These clustering results further support the broad regulatory effect of GA on apoptosis-related pathways, with its influence exhibiting stage-specific characteristics during development. Notably, although several clusters were associated with apoptotic processes and their regulation (especially extrinsic and development-related apoptotic pathways), no enhancement of apoptotic execution was observed at the protein level in our previous experiments (Fig. 5, *E*–*H*). Gene set enrichment analysis (GSEA) further revealed that apoptosis-related pathways were significantly enriched in both CPCs (normalized enrichment score = 1.1253, *p* < 0.05) and early CMs (normalized enrichment score = 1.1624, *p* < 0.05), with slightly higher enrichment in the latter stage (Fig. 6*G*). Notably, analysis of the leading-edge genes driving this enrichment revealed that they predominantly consisted of antiapoptotic factors (Fig. 6*F*), explaining the apparent paradox of an “enriched” apoptotic signature coinciding with a reduction in cell death. Further analysis of the leading-edge genes from the GSEA apoptosis-related pathways showed that several antiapoptotic genes, such as *MCL1*, *XIAP*, *BIRC5*, *API5*, *BCL2*, and *BCL2L1*, were significantly upregulated upon GA treatment (Fig. 6*F*). Combined with the previously observed reduction in apoptotic execution at the protein level (Fig. 5, *E*–*H*) and the upregulation of antiapoptotic genes such as *NONO*, *CLU*, and *SOD1* (Fig. 6*C*), these findings suggest that GA orchestrates multiple antiapoptotic mechanisms at both the transcriptional and protein levels. This indicates that GA may participate in CM fate determination and developmental remodeling through the precise spatiotemporal regulation of apoptosis-related gene expression during cardiac differentiation.

In addition, following GA supplementation, the expression of cardiomyogenesis-related genes was significantly upregulated at the cardiac progenitor and early CM stages (Fig. 6*D*). Key transcription factors involved in CM development, including *NKX2-5*, *TBX5*, *TBX3*, *GATA3*, *GATA4*, and *ISL1*, were also markedly elevated (Fig. 6*E*). These findings are consistent with observations from flow cytometry and beating cell assays (Fig. 3*B*, Videos S1, and S2). Kyoto Encyclopedia of Genes and Genomes (KEGG) and GO enrichment analyses of differentially expressed genes in CPCs (Fig. 6*H*) revealed that in addition to regulating apoptosis and CM differentiation-related pathways, GA treatment also resulted in significant enrichment of pathways related to "signaling pathways regulating pluripotency of stem cells," "ATP-dependent chromatin remodeling," and "cytoskeleton in muscle cells," while also affecting epigenetic processes including RNA splicing and mRNA processing. Moreover, GA supplementation influenced amino acid metabolism during cardiac progenitor differentiation, suggesting its potential involvement in metabolic remodeling during CM fate determination.

In summary, GA orchestrates the spatiotemporal regulation of apoptosis-related gene expression during cardiac differentiation while concurrently activating antiapoptotic mechanisms and key cardiomyogenic programs. This dual role promotes CM differentiation while sustaining cell survival, highlighting its dual role in the regulation of cardiac development.

### Molecular docking predictions

Reverse pharmacophore matching of GA was performed using PharmMapper and DrugCLIP (33, 34), and the resulting potential target proteins are listed in Tables S2 and S3. With GO/KEGG enrichment analysis (Table S4), we defined 175 potential target proteins associated with apoptosis, cell proliferation, and the oxytocin signaling pathway. Considering both the docking scores and a composite binding stability score (incorporating hydrogen bonds, hydrophobic contacts, and other terms), we selected the following proteins for further evaluation: JADE1, IFT80, SEH1L, CYCS (CYC), CACNA1C, CYFIP2, ERCC2, SLC9A1, and PDGFA. Given the critical role of CYC in intrinsic apoptosis (35), it was chosen for further analysis.

**Figure 7:**
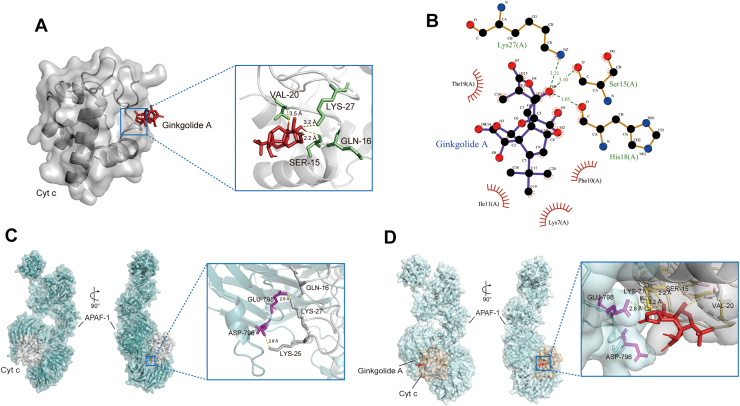
**Molecular docking predicts ginkgolide A binding to cytochrome c and APAF-1.***A*, predicted binding pose of ginkgolide A (*red*) to cytochrome c (*gray*), as calculated by AutoDock Vina. GA interacts with cytochrome c residues Lys27 and Ser15(highlighted as *green sticks*). Hydrogen bond distances are labeled in Ångströms. *B*, two-dimensional interaction map of ginkgolide A bound to cytochrome c. Hydrogen bonds are depicted as *green dashed lines* with donor–acceptor distances labeled. Hydrophobic contacts are indicated by *red arc symbols* with radiating spokes pointing to neighboring residues. *C*, structural model of the cytochrome c (*gray*)/APAF-1 (*cyan*) complex predicted by AlphaFold 3. Key interface residues of cytochrome c (Lys25, Lys27) are highlighted as *purple sticks*, juxtaposed against a complementary acidic cluster on APAF-1 (Asp796, Glu798). Hydrogen bond distances are labeled in Ångströms. *D*, structural superimposition of the ginkgolide A–cytochrome c docking complex (cytochrome c in *orange* and ginkgolide A in *red*) and the CYC–APAF-1 binding model (cytochrome c in *gray* and APAF-1 in *cyan*). The GA-bound complex was superimposed onto the CYC–APAF-1 complex in PyMOL by aligning on the CYC backbone. GA is displayed as *red sticks*. Key residues at the overlapping interface—Lys27, Val20, and Ser15 of CYC—are displayed as *orange sticks* and labeled. Hydrogen bonds are depicted as *dashed lines* with distances labeled in Ångströms. APAF-1, apoptotic protease activating factor 1; GA, ginkgolide A; CYC, cytochrome c.

Molecular docking between GA and CYC was carried out using AutoDock Vina (36) (Fig. 7*A*). The prediction suggests a potential interaction that GA primarily forms hydrogen bonds through its carbonyl oxygen (O9) at C10 with the side chain of Lys27 and Ser15 with a distance of 3.5 Å and 2.2 Å respectively, potentially perturbing the original hydrogen bond network of CYC and altering its conformation. LigPlot + analysis further confirmed the formation of hydrogen bonds: one between the O9 hydroxyl oxygen of GA and the terminal side-chain ammonium nitrogen of Lys27 (3.21 Å), and another between the C10 hydroxyl hydrogen of GA and the O atom of Ser15 (3.05 Å) (Fig. 7*B*). In addition, hydrophobic contacts were observed with residues Lys7, Phe10, Ile11, and Thr19. Transient polar interactions involving residue Val20 were also noted in the 3D model, although they did not satisfy the stringent HBPLUS criteria for stable hydrogen bonds.

CYC is known to trigger apoptosome assembly by binding to apoptotic peptidase activating factor-1 (APAF-1) (36). We therefore employed AlphaFold3 to predict the structure of the CYC-APAF-1 complex (Figs. 7*C* and S1*D*). The predicted interface metrics (ipTM = 0.72, pTM = 0.64) and high predicted confidence (pLDDT > 90 for key regions) suggest a reliable model. Previous studies have identified residues 7, 25, 39, 62 to 65, and 72 of CYC as critical for its binding to APAF-1 (35, 37). Consistent with this, our docking analysis also identified residues 25, 27, 39, 62 to 65, and 72 as part of the binding interface.

The predicted model suggests a stable hydrogen bond interaction network at the APAF-1/CYC interface (Fig. 7*C*). Specifically, a cluster of acidic residues (Asp796 and Glu798) in the linker region between the APAF-1 CARD and nucleotide-binding domains forms multiple, directionally specific hydrogen bonds and potential salt bridges with a cluster of polar/basic residues (Gln16, Lys25, Lys27) on the Ω-loop C of CYC. This electrostatic complementarity provides binding specificity and contributes significantly to affinity, suggesting a conserved molecular recognition hot spot.

The docking results suggest that GA may acts as a competitive inhibitor by binding at or near the CYC/APAF-1 interaction interface (Fig. 7*D*). The predicted binding free energy for this complex is −5.4 kcal/mol, indicating a favorable interaction. Specifically, the model predicts hydrogen bonds formation between the carbonyl oxygen of GA and the side-chain ammonium group of Lys27 as well as the hydroxyl group of Ser15 on CYC, while the existing hydrophobic contacts are also disturbed by GA binding. This interaction is likely to disrupt the electrostatic and hydrogen-bonding network that is essential for interface stability, thereby reducing the binding affinity of APAF-1 for CYC and blocking subsequent apoptosis signal transduction.

## Discussion

*G. biloba* leaf extract is a traditional Asian herbal medicine, which is clinically used in the prevention and treatment of cardiovascular diseases (38, 39). Its heart-protective effects are largely attributed to ginkgolides, the principal bioactive constituents (40). Extensive research indicates that these ginkgolides can alleviate inflammation and apoptosis in neural cells and improve cardiac function by reducing endoplasmic reticulum stress (29, 30, 41). However, their molecular and cellular effects on CMs—particularly during stem cell differentiation—remain unclear. To address this gap, we established a PSC differentiation platform incorporating a CM-specific reporter. Using this system, we discovered that GA inhibits apoptosis during CM differentiation. Furthermore, computational docking analysis predicted CYC as its likely target, revealing a potential binding site at the protein-protein interface critical for apoptosis initiation.

The efficiency of many stem cell-related biomanufacturing processes is constrained by cell quality and culture conditions (42, 43, 44). Especially, when differentiating PSCs into three germ layer lineages, assessing the differentiation potential of stem cells is critical. This is often inferred indirectly through metrics such as overall cell culture quality and the expression levels of pluripotency genes (45). Indeed, the quality of the starting stem cell population is a fundamental determinant of differentiation success, making the optimization of culture conditions a central strategy for improving induction efficiency. For instance, the genetic knockdown of proapoptotic genes *BAK* and *BAX* has been shown to enhance the survival and therapeutic efficacy of mesenchymal stem cells by attenuating apoptosis (46). Likewise, supplementing cultures with bovine serum albumin (BSA) can improve cellular tolerance to differentiation-inducing factors, thereby increasing differentiation capacity from a culture quality standpoint (45). Based on this rationale, we selected CM differentiation—a process with clear, observable functional outcomes—as a direct and relevant indicator to assess the impact of GA on stem cell differentiation potential.

To screen for effective ginkgolide compounds with significant cellular effects, we designed and constructed a CM reporter system that indicates CM formation *via* fluorescence (Figs. 2, *A* and *D* and S1, *A* and *B*). Given that previous reports used ginkgolide concentrations between 30 to 150 μM (28, 30, 41), we tested GA, GB, and GK at 50 μM and 100 μM during CM differentiation. Using beating activity and reporter-based efficiency as metrics (Fig. 3*B*), we found that GA and GB significantly enhanced induction efficiency (Fig. 3, *B* and *C*). Notably, with GA treatment, the first beating could consistently occur on Day 5 (Video S1).

To determine the optimal concentration, we tested 0, 5, 20, 100, 150, and 200 μM GA (Fig. S1*C*). Beating on Day 5 occurred at 100 μM and 150 μM (Videos S1 and S2), and NKX2-5 protein expression was notably increased on Day 5 with GA treatment (Fig. 4, *A* and *B*). Similar to findings in neural cells (47, 48, 49), GA exhibited antiapoptotic effects during CM differentiation. We assessed Caspase-3 activity and Annexin V (Fig. 5, *A* and *B*), cardiac mesoderm marker gene expression (Day 4, Fig. 5*C*), CPC marker gene expression (Day 5, Fig. 5*D*), and apoptosis-related protein expression in undifferentiated stem cells and Day 5 cells treated with 0, 20, 100, 150, and 200 μM GA (Fig. 5, *E*–*H*). Results indicated that apoptosis occurs during differentiation and GA effectively inhibits it. The enhanced differentiation efficiency is likely due to improved culture quality *via* reduced apoptosis, thereby boosting the differentiation capacity of PSCs. Interestingly, at the high dose of 200 μM, GA completely blocked differentiation at the cardiac mesoderm stage, indicating that a basal level of apoptosis is necessary for functional CM differentiation. This is consistent with the notion that developmental apoptosis plays a constructive role in tissue morphogenesis and cell fate specification (50, 51).

We next performed RNA-Seq at three core time points of CMs differentiation: cardiac mesoderm (Day 4), cardiac progenitor (Day 5), and early CMs (Day 6) with or without GA treatment of 150 μM GA. Pearson correlation and PCA revealed that GA addition did not exert effects at all stages; especially the PSC stage, the GA addition had no significant impact on the transcriptome (Fig. 6, *A* and *B*). However, GA significantly altered gene expression at the cardiac progenitor and early CM stages (Fig. 6, *D* and *E*). This stage-specific effect suggests that GA does not act as a broad transcriptional modulator but rather as a specific regulator during cell fate specification, a period when apoptosis is particularly critical (52). Consistent with our phenotypic observations, GO enrichment analysis of differentially expressed genes identified multiple clusters associated with apoptotic processes, in which antiapoptotic genes were notably upregulated (Fig. 6, *C* and *F*). GSEA further confirmed that apoptosis-related pathways were significantly enriched in GA-treated CPCs and early CMs, with a slight increase in enrichment at the later stage (Fig. 6*G*). Combined with protein analysis by Western blotting (Fig. 5, *E*–*H*), these transcriptomic results indicate that GA exerts its antiapoptotic effect at least in part through the upregulation of antiapoptotic genes. This also reveals that GA has a greater impact on differentiation-induced apoptosis, which peaks during the progenitor-to-CM transition (53). Beyond apoptosis, KEGG and GO enrichment analyses revealed that GA treatment also modulated pathways related to some epigenetic regulation terms (Fig. 6*H*), which is critical for the mechanical and functional maturation of CMs (54). In addition, the observed influence on amino acid metabolism hints at a potential role for GA in metabolic remodeling during cardiac differentiation—a process increasingly recognized as a key driver of cell fate decisions (55, 56).

To further elucidate the molecular basis of GA’s antiapoptotic effect, we performed molecular docking to identify potential targets (Table S1–S3). Based on predictions and enrichment results, GA shows high potential as a competitive inhibitor targeting CYC. The core finding from our molecular docking and binding free energy calculations is that GA binds stably at the known CYC/APAF-1 interface with a predicted affinity of −5.4 kcal/mol. Although this interaction is thermodynamically weaker than the intrinsic binding between APAF-1 and CYC (estimated ΔG ≈ −10.8 kcal/mol) (57, 58), the binding of GA at or near the CYC/APAF-1 interface is functionally critical. GA is predicted to form specific hydrogen bonds and hydrophobic interactions with CYC (Fig. 7, *A* and *B*). These interactions could perturb the precise electrostatic and hydrogen-bonding network between APAF-1 and CYC, thereby reducing their recognition and binding affinity and ultimately interfering with apoptotic signal transduction.

The interaction between CYC and APAF-1 is characterized by an exceptionally high binding affinity, with a reported association constant exceeding 10^11^ M^-1^ under ultralow salt conditions (corresponding to a binding free energy of approximately −15.6 kcal/mol) (58). Such tight binding is consistent with a switch-like mechanism that is crucial for the apoptosis initiation. This extreme affinity context suggests that the primary window for GA to exert its effect is likely prior to the commitment of CYC to APAF-1 binding. This interpretation aligns with our experimental design, where GA treatment was applied from the onset of differentiation. Furthermore, it is important to note that predicted binding affinities often differ from experimentally determined values (59). Despite this inherent discrepancy, the mechanistic evidence demonstrating GA's inhibition of apoptotic execution (Fig. 5, *E*–*H*) validates that it produces a functional outcome consistent with the computational model.

Consistently, downstream proteins of the CYC/APAF-1 pathway, Cleaved Caspase-9, Cleaved Caspase-3, and Cleaved PARP, decreased upon GA addition (Fig. 5, *E*–*H*). This supports the notion that GA largely exerts its antiapoptotic activity through a mechanism consistent with the potential inhibition of APAF-1/CYC binding, subsequently improving stem cell culture quality during induction and enhancing differentiation efficiency. Although the *in silico* model places GA near the critical interface and suggests a plausible steric hindrance mechanism, confirming direct target engagement and the exact binding pose will require future biophysical studies. Notably, while Cleaved Caspase-9 and Cleaved Caspase-3 decreased, total Caspase-9 and Caspase-3 protein levels increased (Fig. 5, *E*–*K*). This indicates GA effectively inhibits the caspase activation cascade, leading to intracellular accumulation of inactive procaspases and full-length PARP, which explains elevated total protein levels on Western blots.

How the transcriptomic changes relate to the predicted disruption of CYC/APAF-1 binding remains to be fully resolved. The elevated expression of antiapoptotic genes detected by RNA-Seq does not, in itself, constitute evidence for direct transcriptional activation by GA. A more economical reading of the data is that GA impairs CYC/APAF-1 complex formation, thereby blunting the caspase amplification cascade (Fig. 5, *E*–*G*) and lowering the overall propensity for apoptosis. When apoptotic pressure subsides, the transcriptome of differentiating cardiac progenitors may drift toward a prosurvival configuration. One mechanism that could contribute to such a shift is the relief of caspase mediated cleavage or functional inactivation of lineage specifying transcription factors (60). Notably, sustained *MCL1* and *XIAP* levels have been shown to safeguard the cardiac transcriptional network during commitment (61). Within this framework, the docking predictions and the transcriptomic signature do not represent parallel or competing mechanisms. Instead, they likely describe a hierarchical sequence: an initial perturbation at the CYC/APAF-1 protein–protein interface is followed by a secondary consolidation of the prosurvival milieu, the latter being reinforced through sustained anti apoptotic gene expression during cardiac differentiation.

Specifically, employing GA as a novel, safe, plant-derived culture additive holds promise for addressing the seed cell culture quality issue in the biology manufacturing related to stem cell culture. On the one hand, GA can enhance cell viability and function during large-scale expansion; on the other hand, it can improve the efficiency and uniformity of directed differentiation into functional cells. By reducing cell loss during bioreactor culture and differentiation, GA can directly contribute to lowering production costs and increasing final product yield. Furthermore, the purified compound GA is derived from *G. biloba*, a traditional medicinal and food homologous plant, which provides a favorable safety foundation for its use as a food additive component (62). This gives it unique application potential and regulatory acceptance advantages over synthetic chemicals in medical-grade cell culture systems. Therefore, this work not only proposes a model for the mechanism of GA in cell fate regulation by phenotypic and computational evidence, but also provides a valuable candidate tool and scientific basis for developing efficient and sustainable cellular agriculture technology systems based on natural products.

Collectively, the transcriptomic data reveal that GA acts not as a simple antiapoptotic agent, but as a multifunctional regulator that integrates survival signals with lineage-specific transcriptional programs. By coordinating the upregulation of antiapoptotic genes with the activation of core cardiogenic transcription factors, GA creates a prosurvival, prodifferentiation microenvironment that enhances CM yield without compromising cell identity. This study thus positions GA as a promising chemical tool for both mechanistic studies of stem cell differentiation and practical applications in cell-based manufacturing.

## Experimental procedures

### Cell lines and cell culture

The hPSC line (CB0003) used in this study was obtained from the National Stem Cell Resource Center (China) (63). Cells were maintained in 2D culture on hESC-qualified Matrigel (Corning, cat. no. 354277) using StemFlex medium (Gibco, cat. no. A3349401), with medium changes every other day. Cells were cultured at 37 °C with 5% CO_2_ and passaged every 3 to 5 days using Versene solution (Gibco, cat. no. 15040066) (5 min incubation at 37 °C). For passaging, StemFlex medium supplemented with 10 μM Y27632 (MCE, cat. no. HY-10071) was used for the first 24 h, followed by regular StemFlex medium. The cell splitting ratio was between 1:15 and 1:25. All cell lines tested negative for *mycoplasma* by PCR.

### CM differentiation from pluripotent stem cells

Based on the CM differentiation protocol for hPSCs (45, 64), we introduced modifications. RPMI 1640 (Gibco, cat. no. 11875119) was used as the basal medium, supplemented with 2% B27 minus insulin (Gibco, cat. no. A1895601), 65 μg/ml L-ascorbic acid 2-phosphate (Sigma-Aldrich, cat. no. 49752), and 1% penicillin-streptomycin (Gibco, cat. no. 15140122) to constitute the induction medium. Differentiation was initiated when hESC confluence reached 70 to 80%. On Day 0, the induction medium was supplemented with 6 μM CHIR99021 (MCE, cat. no. HY-10182), 5 ng/ml fibroblast growth factor-basic (SinoBio, cat. no. 10014-HNAE), 5 ng/ml BMP4 (PeproTech, cat. no. 120–05 ET), and 5 ng/ml Activin A (SinoBio, cat. no. 10429-HNAH). On Day 2, the medium was changed to induction medium supplemented with 1 μM IWR-1 (Sigma-Aldrich, cat. no. I0161) and 0.5 μM retinoic acid (MCE, cat. no. HY-14649). After Day 4 induction, the medium was replaced with induction medium supplemented with 5 ng/ml BMP4 and 10 ng/ml fibroblast growth factor 10 (SinoBio, cat. no. 10573-HNAE). On Day 6, the medium was changed to CM maintenance medium, consisting of RPMI 1640 supplemented with 2% B27 (Gibco, cat. no. 17502048), 65 μg/ml L-ascorbic acid, and 1% penicillin-streptomycin. On Day 8, a CM selection medium consisting of glucose-free RPMI 1640, 2% B27, 65 μg/ml L-ascorbic acid 2-phosphate, 1% penicillin-streptomycin, and 5 mM sodium L-lactate (Sigma-Aldrich, cat. no. L7022) was used for 2 days. The CMs were then kept in the maintenance medium.

For screening the effects of GA (MCE, cat. no. HY-B0355), GB (MCE, cat. no. HY-N0784), and GK (MCE, cat. no. HY-N4176) on differentiation efficiency, compounds were added at concentrations of 50 μM and 100 μM throughout the entire differentiation process based on the concentration reported before (65). For subsequent functional validation of GA, a concentration-response assay was performed. Differentiating cells were treated with GA at 0 μM (dimethyl sulfoxide vehicle control, Sigma-Aldrich, cat. no. V900090), 5 μM, 20 μM, 100 μM, 150 μM, and 200 μM throughout differentiation.

### Reporter cell line construction

The human *TNNT2* gene sequence was obtained from NCBI. The sgRNA sequence targeting the region just before the stop codon (Table S1) was designed using the CRISPR website from Zhang Feng's team (https://crispor.gi.ucsc.edu/). After oligo annealing, the double strand DNA was ligated into the PX459 vector using T4 ligase (Thermo Fisher Scientific, cat. no. EL0012). The donor vector was constructed *via* four-fragment homologous recombination by NovoRec plus One step PCR Cloning Kit 2.0 (Novoprotein, cat. no. NR006). Homology arm sequences upstream and downstream of the stop codon were amplified from the 293T cell genome. The donor vector backbone and bacterial amplification backbone were obtained by restriction enzyme digestion and gel extraction. Fragments were ligated using a homologous recombination enzyme and verified by sequencing. The two plasmids were mixed at a 1:1 M ratio and transfected into hPSCs using Lipofectamine Stem reagent (Thermo Fisher Scientific, cat. no. STEM00015).

A puromycin selection preexperiment was performed on hPSCs to determine the optimal concentration (0.1, 0.5, 1, 5, 10, 20, 50, 100 μg/ml for 24 h, observing cell death). The optimal concentration was determined to be 5 μg/ml. Posttransfection cells were selected with puromycin for 48 h. After selection, cells were washed twice with Dulbecco's phosphate-buffered saline without calcium and magnesium (Gibco, cat. no. 14190144), and single clones were picked under a microscope. When clones expanded sufficiently, they were passaged. Part of the cells were collected for genomic DNA extraction. PCR was performed using primers Hum-TNNT2-Down-RA and hum-TNNT2-UP-LA (Table S1). PCR products were separated by agarose gel electrophoresis and knock in was confirmed by Sanger sequencing.

### qRT-PCR analysis

The procedure was performed as previously described (66). Cells from differentiation days 2, 4, 6, and 8 were digested and collected using EDTA-0.25% trypsin (Gibco, cat. no. 25200056), neutralized with high-glucose DMEM (Gibco, cat. no. C11995500BT) containing 10% fetal bovine serum (FBS) (Vistech, cat. no. SE200-ES), and centrifuged at 300 g for 5 min to collect cell pellets. Total RNA was extracted using RNA-iso Plus (Takara, cat. no. 9108), dissolved in DEPC-treated water (Beyotime, cat. no. R0021), and reverse transcribed using the HiScript IV first Strand cDNA Synthesis Kit (Vazyme, cat. no. R323). qPCR was performed using ChamQ SYBR qPCR Master Mix (Vazyme, cat. no. Q311) on a Bio-Rad CFX96 system with the following program: 95 °C for 10 s, 60 °C for 30 s, 72 °C for 30 s, for 40 cycles. Primer sequences are listed in Table S1.

### Western blotting

Experimental procedures for Western blotting followed the methods established in our prior work (67). For protein extraction, cells were collected and lysed in the radioimmunoprecipitation assay buffer (Beyotime, cat. no. P0013 B) with 1% PMSF (Solarbio, cat. no. P0100) and 1% protease inhibitor cocktail (Solarbio, cat. no. IKM1010) on ice for 30 min, with vortexing every 5 min until the lysate cleared. Then the lysate was boiled with 5 × SDS loading buffer (Beyotime, cat. no. P0015) at 100 °C for 10 min. After aliquoting, the samples were subjected to the 10% or 15% SDS-PAGE. The SDS-PAGE gel was prepared by one-step PAGE gel kit (Yeasen, cat. no. 20325ES62) according to the manufacturer’s instructions. The protein marker is 10 to 180 kDa StarRuler Color Prestained Protein Marker (Genestar, cat. no. M221). Electrophoresis was performed at 70 V for 30 min followed by 120 V until completion. Proteins were transferred to methanol-activated 0.45 μm polyvinylidene fluoride membranes (Sigma-Aldrich, cat. no. IPVH00010) at 400 mA constant current. Membranes were blocked with 8% nonfat milk (Solarbio, cat. no. D8340) in tris-buffered saline with tween-20 (TBST) (Solarbio, cat. no. T1081) for 2 h, incubated with primary antibody at 4 °C overnight, washed three times with TBST (10 min each), incubated with secondary antibody for 1 h at RT, and washed again three times with TBST (15 min each). Signals were visualized using ECL Detection Reagent (Mishushengwu, cat. no. MI00607) and imaged with a gel documentation system (Tanon). Anti-PARP (cat. no. T56850), anti-Caspase3 (cat. no. T40044), anti-Caspase9 (cat. no. T40046), anti-BCL2 (cat. no. T40056) and anti-BAX (cat. no. T40051) antibodies were purchased from Abmart. HRP-conjugated anti-rabbit (cat. no. BM2006) and anti-mouse (cat. no. BA1075) secondary antibodies for Western blotting were from Boster. All antibodies and blocking solution were prepared in TBST.

### Flow cytometry

For the reporter cell line, cells were digested with Accutase (Stemcell, cat. no. 07920), washed with D-Hanks' solution (Gibco, cat. no. 14025092) containing 5% FBS (Vistech), and analyzed directly. For intracellular protein detection, digested cells were first fixed with 4% paraformaldehyde (Solarbio, cat. no. P1110) and permeabilized with 0.1% Triton X-100 (Solarbio, cat. no. T8200). After blocking with 5% BSA (Sigma-Aldrich, cat. no. V900933), cells were incubated with primary antibodies: TNNT2 antibody (MA5-12960, Invitrogen) and NKX2-5 antibody (8792, CST) diluted 1:200 in PBS (Gibco) containing 0.5% BSA, followed by Alexa Fluor 568 Goat anti-Mouse IgG (A-11036, Thermo Fisher Scientific), Alexa Fluor 647-labeled goat anti-rabbit IgG secondary antibody (cat. no. A-21245, Thermo Fisher Scientific), and Alexa Fluor 647-labeled goat anti-mouse IgG secondary antibody (cat. no. A-21235 Thermo Fisher Scientific) diluted 1:1000 in PBS containing 0.5% BSA.

For apoptosis detection, the Live Cell Caspase-3 Activity and Annexin V Apoptosis Detection Kit (Beyotime, cat. no. C1077M) was used. All procedures were performed according to the manufacturer’s instructions.

### Transmission electron microscopy

Cell samples were fixed with 3% glutaraldehyde (in 0.1 M phosphate buffer, pH 7.4) at 4 °C, followed by postfixation with 1% osmium tetroxide. After dehydration through a graded acetone series, the samples were infiltrated and embedded in Epon 812 resin. Semithin sections were stained with methylene blue for light microscopy. Ultrathin sections (approximately 70 nm thick) were cut using a diamond knife, double-stained with uranyl acetate and lead citrate, and subsequently examined under a JEM-1400-FLASH transmission electron microscope.

### Immunofluorescence staining

Immunofluorescence staining was performed as previously described (68). Cells were washed with PBS, fixed with 4% paraformaldehyde, permeabilized with 0.1% Triton X-100, washed, and blocked with PBS containing 10% FBS (Vistech). Cells were incubated overnight at 4 °C with each target antibody at the manufacturer’s recommended dilution. After washing with PBS, secondary fluorescence-labeled antibody was applied at 1:200 dilution and incubated at RT for 1 h. Finally, nuclei were stained with 4′,6-diamidino-2-phenylindole (Solarbio, cat. no. C0060) diluted 1:1000. Images were captured using a Thermo Fisher Scientific M5000 fluorescence microscope . Anti-TNNT2 (cat. no. MA5-12960), Alexa Fluor 568 Goat anti-Rabbit secondary antibody (cat. no. A-11036), Alexa Fluor 488-labeled goat anti-rabbit IgG secondary antibody (cat. no. A-11008) was from Invitrogen, Goat anti-mouse IgG H&L Alexa Fluor 488 (cat. no. ab150113) was from Abcam, anti-NKX2-5 (cat. no. 8792) from Cell Signaling Technology, anti-MYL2 (cat. no. 10906-1-AP), anti-ISL1 (cat. no.15661-1-AP), and anti-MESP1 (cat. no. 32304-1-AP) was from Proteintech.

### RNA-Seq

Total RNA was extracted from GA-treated (150 μM) and control PSC-derived cardiac mesoderm (Day 4), CPCs (Day 5), and early CMs (Day 6) using RNAiso Plus (Takara, cat. no. 9108). OD260/280 is between 1.18 and 2.2, OD260/230 > 2.0, RIN ≥ 6.5, 28S:18S ≥ 1.0, to ensure that there is no degradation of RNA and no contamination. The mRNA with polyadenylate was specifically captured by two rounds of purification using oligo(dT) magnetic beads (Dynabeads Oligo (dT), Thermo Fisher Scientific, cat. no. 25–61005). The captured mRNAs were fragmented using the Magnesium Ion Interruption Kit (NEBNextR Magnesium RNA Fragmentation Module, cat. no. E6150S). The fragmented RNA was passed through reverse transcriptase (Invitrogen SuperScriptTM II Reverse Transcriptase, CA, cat. no. 1896649) to synthesize cDNA. The second strand was then synthesized using *E. coli* DNA polymerase I (NEB, cat. no. m0209) and RNase H (NEB, cat. no. m0297) to convert the DNA-RNA hybrids into double strands DNA, while labeling the duplexes with dUTP Solution (Thermo Fisher Scientific, cat. no. R0133) to complement the ends of double-stranded DNA to flat ends. The second strand was digested with UDG enzyme (NEB, cat. no. m0280) and then a strand-specific library was constructed by PCR. The average insert size for the final cDNA libraries were 300 ± 50 bp. Illumina NovaseqTM 6000 (LC Bio Technology CO, Ltd) was used to perform bipartite sequencing according to the standard operation, and the sequencing mode was PE150.

### Molecular docking

We performed reverse pharmacophore matching for GA (PubChem CID: 9909368) using both PharmMapper and DrugCLIP (33, 34, 69, 70). Potential target proteins identified from PharmMapper (Fit Score ≥ 2.0) and DrugCLIP (top 1% by predicted score) were subsequently subjected to GO and KEGG pathway enrichment analysis using the KOBAS database (71). Molecular docking between GA and the potential target CYC (PDB ID: 3ZOO) was performed using AutoDock Vina (version 1.2.6) (36, 72, 73). Docking results were filtered based on calculated affinity ≤ −5.0 kcal/mol (74). Binding site analysis was performed using the PyMOL Molecular Graphics System (version 1.3). Based on the predicted interaction between APAF-1 (PDB ID: 1Z6T) and CYC in the apoptosis signaling pathway by AlphaFold3 (75). The predicted binding poses of GA on CYC were analyzed and compared. The predicted binding affinity for each pose was estimated using AutoDock Vina with default settings, which provides a docking score that correlates with binding strength. Hydrogen bonds were identified and validated using LigPlot+ (HBPLUS algorithm, Version 2.1) with default geometric criteria settings (76, 77). PyMOL was used for initial visual inspection and to assess overall binding pose.

### Statistical analysis

In this study, transcriptomics data were analyzed and plotted by R (V4.4.3) (78), the following R packages were used for the analysis: DESeq2 (version 1.46.0), org.Hs.e.g.db (version 3.19.1), corrplot (version 0.95), ggplot2 (version 3.5.1), and fgsea (version 1.32.4). And the quantification of Western Blotting was analyzed by imageJ (Version 1.54f). Each cell experiment was performed with at least three replicates. Results are presented as mean ± SD. GraphPad Prism (version 8.3.0) was used for graphing and statistical analysis. Differences among multiple groups were analyzed by one-way ANOVA, followed by Tukey's multiple comparisons test for pairwise comparisons. A *p*-value < 0.05 was considered statistically significant.

## Data availability

The raw RNA-Seq data reported in this study are available from the NCBI database (SRA Accession: PRJNA1184182, PRJNA1464420). All data are available on request.

## Supporting information

This article contains supporting information. (33, 34, 66, 69, 70, 72, 73, 75).

## Conflict of interest

The authors declare that they have no conflicts of interest with the contents of this article.

## References

[1] Severino P., D’Amato A., Pucci M., Infusino F., Adamo F., Birtolo L.I. (2020). Ischemic heart disease pathophysiology paradigms overview: from plaque activation to microvascular dysfunction. Int. J. Mol. Sci..

[2] Yang J., Lei W., Xiao Y., Tan S., Yang J., Lin Y. (2024). Generation of human vascularized and chambered cardiac organoids for cardiac disease modelling and drug evaluation. Cell Prolif..

[3] Entcheva E., Kay M.W. (2021). Cardiac optogenetics: a decade of enlightenment. Nat. Rev. Cardiol..

[4] Lee J.H., Protze S.I., Laksman Z., Backx P.H., Keller G.M. (2017). Human pluripotent stem cell-derived atrial and ventricular cardiomyocytes develop from distinct mesoderm populations. Cell Stem Cell.

[5] Floy M.E., Shabnam F., Simmons A.D., Bhute V.J., Jin G., Friedrich W.A. (2022). Advances in manufacturing cardiomyocytes from human pluripotent stem cells. Annu. Rev. Chem. Biomol. Eng..

[6] Kim H., Kamm R.D., Vunjak-Novakovic G., Wu J.C. (2022). Progress in multicellular human cardiac organoids for clinical applications. Cell Stem Cell.

[7] He R., Wang Z., Cui M., Liu S., Wu W., Chen M. (2021). HIF1A Alleviates compression-induced apoptosis of nucleus pulposus derived stem cells via upregulating autophagy. Autophagy.

[8] Fu Y., Sui B., Xiang L., Yan X., Wu D., Shi S. (2021). Emerging understanding of apoptosis in mediating mesenchymal stem cell therapy. Cell Death Dis..

[9] Xiaoyan L., Li C., Liu T., Ke H., Gong X., Wang Q. (2018). Chemical analysis, pharmacological activity and process optimization of the proportion of bilobalide and ginkgolides in Ginkgo biloba extract. J. Pharm. Biomed. Anal..

[10] Sarkar C., Quispe C., Jamaddar S., Hossain R., Ray P., Mondal M. (2020). Therapeutic promises of ginkgolide A: a literature-based review. Biomed. Pharmacother..

[11] Wang S., Ouyang B., Aa J., Geng J., Fei F., Wang P. (2016). Pharmacokinetics and tissue distribution of ginkgolide A, ginkgolide B, and ginkgolide K after intravenous infusion of ginkgo diterpene lactones in a rat model. J. Pharm. Biomed. Anal..

[12] Kuo L.-C., Song Y.-Q., Yao C.-A., Cheng I.H., Chien C.-T., Lee G.-C. (2019). Ginkgolide A prevents the Amyloid-β-Induced depolarization of cortical neurons. J. Agric. Food Chem..

[13] Chen Y., Wang C., Hu M., Pan J., Chen J., Duan P. (2012). Effects of ginkgolide A on Okadaic acid-induced tau hyperphosphorylation and the PI3K-Akt signaling pathway in N2a cells. Planta Med..

[14] Park S., Park M., Lee H.-J. (2025). Ginkgolide A enhances cognition and reduces amyloid-β by activating autophagy in the murine 5xFAD Alzheimer’s disease model. Biomed. Pharmacother..

[15] Duan H., Zhang Q., Liu J., Li R., Wang D., Peng W. (2021). Suppression of apoptosis in vascular endothelial cell, the promising way for natural medicines to treat atherosclerosis. Pharmacol. Res..

[16] Bertheloot D., Latz E., Franklin B.S. (2021). Necroptosis, pyroptosis and apoptosis: an intricate game of cell death. Cell Mol. Immunol..

[17] Burridge P.W., Matsa E., Shukla P., Lin Z.C., Churko J.M., Ebert A.D. (2014). Chemically defined generation of human cardiomyocytes. Nat. Methods.

[18] Lian X., Bao X., Zilberter M., Westman M., Fisahn A., Hsiao C. (2015). Chemically defined, albumin-free human cardiomyocyte generation. Nat. Methods.

[19] Bao X., Lian X., Qian T., Bhute V.J., Han T., Palecek S.P. (2017). Directed differentiation and long-term maintenance of epicardial cells derived from human pluripotent stem cells under fully defined conditions. Nat. Protoc..

[20] Stefanovic S., Zaffran S. (2017). Mechanisms of retinoic acid signaling during cardiogenesis. Mech. Development.

[21] Hubert F., Payan S.M., Pelce E., Bouchard L., Sturny R., Lenfant N. (2022). FGF10 promotes cardiac repair through a dual cellular mechanism increasing cardiomyocyte renewal and inhibiting fibrosis. Cardiovasc. Res..

[22] Klaus A., Müller M., Schulz H., Saga Y., Martin J.F., Birchmeier W. (2012). Wnt/β-catenin and Bmp signals control distinct sets of transcription factors in cardiac progenitor cells. Proc. Natl. Acad. Sci. U. S. A..

[23] Jia G., Preussner J., Chen X., Guenther S., Yuan X., Yekelchyk M. (2018). Single cell RNA-seq and ATAC-seq analysis of cardiac progenitor cell transition states and lineage settlement. Nat. Commun..

[24] Thomas D., Cunningham N.J., Shenoy S., Wu J.C. (2022). Human-induced pluripotent stemcells in cardiovascular research: current approaches in cardiac differentiation, maturation strategies, and scalable production. Cardiovasc. Res..

[25] Ding S., Zhang X., Qiu H., Wo J., Zhang F., Na J. (2022). Non-cardiomyocytes in the heart in embryo development, health, and disease, a single-cell perspective. Front. Cell Dev. Biol..

[26] Burridge P.W., Thompson S., Millrod M.A., Weinberg S., Yuan X., Peters A. (2011). A universal system for highly efficient cardiac differentiation of human induced pluripotent stem cells that eliminates interline variability. PLoS One.

[27] Ma S., Liu H., Jiao H., Wang L., Chen L., Liang J. (2012). Neuroprotective effect of ginkgolide K on glutamate-induced cytotoxicity in PC 12 cells via inhibition of ROS generation and Ca(2+) influx. Neurotoxicology..

[28] Hua J., Yin N., Yang B., Zhang J., Ding J., Fan Y. (2017). Ginkgolide B and bilobalide ameliorate neural cell apoptosis in α-synuclein aggregates. Biomed. Pharmacother..

[29] Zhaocheng J., Jinfeng L., Luchang Y., Yequan S., Feng L., Kai W. (2016). Ginkgolide A inhibits lipopolysaccharide-induced inflammatory response in human coronary artery endothelial cells *via* downregulation of TLR4-NF-κB signaling through PI3K/Akt pathway. Pharmazie.

[30] Wang S., Wang Z., Fan Q., Guo J., Galli G., Du G. (2016). Ginkgolide K protects the heart against endoplasmic reticulum stress injury by activating the inositol-requiring enzyme 1α/X box-binding protein-1 pathway. Br. J. Pharmacol..

[31] Feng W., Schriever H., Jiang S., Bais A., Wu H., Kostka D. (2022). Computational profiling of hiPSC-derived heart organoids reveals chamber defects associated with NKX2-5 deficiency. Commun. Biol..

[32] Rashid M., Zadeh L.R., Baradaran B., Molavi O., Ghesmati Z., Sabzichi M. (2021). Up-down regulation of HIF-1α in cancer progression. Gene.

[33] Wang X., Shen Y., Wang S., Li S., Zhang W., Liu X. (2017). PharmMapper 2017 update: a web server for potential drug target identification with a comprehensive target pharmacophore database. Nucleic Acids Res..

[34] Jia Y., Gao B., Tan J., Zheng J., Hong X., Zhu W. (2026). Deep contrastive learning enables genome-wide virtual screening. Science.

[35] Zhou Z., Arroum T., Luo X., Kang R., Lee Y.J., Tang D. (2024). Diverse functions of cytochrome c in cell death and disease. Cell Death Differ..

[36] Eberhardt J., Santos-Martins D., Tillack A.F., Forli S. (2021). AutoDock Vina 1.2.0: new docking methods, expanded force field, and python bindings. J. Chem. Inf. Model..

[37] Yuan S., Topf M., Reubold T.F., Eschenburg S., Akey C.W. (2013). Changes in Apaf-1 conformation that drive apoptosome assembly. Biochemistry.

[38] Gao X., Liu F., Han X., Tang S., Shen D., Zhang J. (2025). A randomized controlled study on the clinical efficacy of Ginkgo Biloba combined with nicorandil in patients with HFmrEF. Int. Heart J..

[39] Karmazyn M., Gan X.T. (2023). Inhibition of myocardial remodeling and heart failure by traditional herbal medications: evidence from Ginseng and ginkgo biloba. Rev. Cardiovasc. Med..

[40] Jaracz S., Stromgaard K., Nakanishi K. (2002). Ginkgolides: selective acetylations, translactonization, and biological evaluation. J. Org. Chem..

[41] Wang X., Jiang C.-M., Wan H.-Y., Wu J.-L., Quan W.-Q., Wu K.-Y. (2014). Neuroprotection against permanent focal cerebral ischemia by ginkgolides A and B is associated with obstruction of the mitochondrial apoptotic pathway via inhibition of c-Jun N-terminal kinase in rats. J. Neurosci. Res..

[42] Mao R., Zhang J., Qin H., Liu Y., Xing Y., Zeng W. (2025). Application progress of bio-manufacturing technology in kidney organoids. Biofabrication.

[43] Bergin A., Carvell J., Butler M. (2022). Applications of bio-capacitance to cell culture manufacturing. Biotechnol. Adv..

[44] Zadpoor A.A. (2017). Design for additive Bio-Manufacturing: from patient-specific medical devices to rationally designed meta-biomaterials. Int. J. Mol. Sci..

[45] Burridge P.W., Holmström A., Wu J.C. (2015). Chemically defined culture and cardiomyocyte differentiation of human pluripotent stem cells. CP Hum. Genet..

[46] Pang S.H.M., D’Rozario J., Mendonca S., Bhuvan T., Payne N.L., Zheng D. (2021). Mesenchymal stromal cell apoptosis is required for their therapeutic function. Nat. Commun..

[47] Zhu L., Li Z., Sheng L., Zhang F., Ji W. (2024). Ginkgolide A attenuated apoptosis via inhibition of oxidative stress in mice with traumatic brain injury. Heliyon.

[48] Chen J., Ou Z., Gao T., Yang Y., Shu A., Xu H. (2022). Ginkgolide B alleviates oxidative stress and ferroptosis by inhibiting GPX4 ubiquitination to improve diabetic nephropathy. Biomed. Pharmacother..

[49] Zhang Y., Miao J.-M. (2018). Ginkgolide K promotes astrocyte proliferation and migration after oxygen-glucose deprivation via inducing protective autophagy through the AMPK/mTOR/ULK1 signaling pathway. Eur. J. Pharmacol..

[50] Kim J.-Y., Cha Y.-G., Cho S.-W., Kim E.-J., Lee M.-J., Lee J.-M. (2006). Inhibition of apoptosis in early tooth development alters tooth shape and size. J. Dent. Res..

[51] Stringer J.M., Alesi L.R., Winship A.L., Hutt K.J. (2023). Beyond apoptosis: evidence of other regulated cell death pathways in the ovary throughout development and life. Hum. Reprod. Update.

[52] Ghose P., Shaham S. (2020). Cell death in animal development. Development.

[53] Wang X., Tao X., Chen P., Jiang P., Li W., Chang H. (2024). MEK inhibition prevents CAR-T cell exhaustion and differentiation via downregulation of c-Fos and JunB. Signal. Transduct. Target. Ther..

[54] Lin C.-Y., Chang Y.-M., Tseng H.-Y., Shih Y.-L., Yeh H.-H., Liao Y.-R. (2023). Epigenetic regulator RNF20 underlies temporal hierarchy of gene expression to regulate postnatal cardiomyocyte polarization. Cell Rep..

[55] Yang X., Rodriguez M.L., Leonard A., Sun L., Fischer K.A., Wang Y. (2019). Fatty acids enhance the maturation of cardiomyocytes derived from human pluripotent stem cells. Stem Cell Rep..

[56] Tang X. (2023). Regenerating the heart by metabolically reprogramming the cardiomyocyte epigenome. Cell Metab..

[57] Purring-Koch C., McLendon G. (2000). Cytochrome c binding to Apaf-1: the effects of dATP and ionic strength. Proc. Natl. Acad. Sci. U. S. A..

[58] Purring C., Zou H., Wang X., McLendon G. (1999). Stoichiometry, free energy, and kinetic aspects of cytochrome *c* : Apaf-1 binding in apoptosis. J. Am. Chem. Soc..

[59] Jereva D., Alov P., Tsakovska I., Angelova M., Atanassova V., Vassilev P. (2022). Application of InterCriteria analysis to assess the performance of scoring functions in molecular docking software packages. Mathematics.

[60] Lüthi A.U., Martin S.J. (2007). The CASBAH: a searchable database of caspase substrates. Cell Death Differ..

[61] Opferman J.T., Kothari A. (2018). Anti-apoptotic BCL-2 family members in development. Cell Death Differ..

[62] Giampieri F., Mazzoni L., Cianciosi D., Alvarez-Suarez J.M., Regolo L., Sánchez-González C. (2022). Organic vs conventional plant-based foods: a review. Food Chem..

[63] Shi Y., Sun Q., Jia F., Xie X., Zhou X., Guo R. (2025). Oncogenic fusions converge on shared mechanisms in initiating astroblastoma. Nature.

[64] Dark N., Cosson M.-V., Tsansizi L.I., Owen T.J., Ferraro E., Francis A.J. (2023). Generation of left ventricle-like cardiomyocytes with improved structural, functional, and metabolic maturity from human pluripotent stem cells. Cell Rep. Methods.

[65] Zhou W., Chai H., Courson A., Lin P.H., Lumsden A.B., Yao Q. (2006). Ginkgolide A attenuates homocysteine-induced endothelial dysfunction in porcine coronary arteries. J. Vasc. Surg..

[66] Wu X., Ni Y., Li W., Yang B., Yang X., Zhu Z. (2024). Rapid conversion of porcine pluripotent stem cells into macrophages with chemically defined conditions. J. Biol. Chem..

[67] Yang D., Chen W., Zhang N., Zhang M., Wu W., Yang L. (2025). UCHL1 regulates adiponectin receptors in Sertoli cells to maintain testicular homeostatic balance. J. Biol. Chem..

[68] Yang X., Wu X., Wang Y., Li W., Wu X., Yuan L. (2024). Induction of lung progenitor cell-like organoids by porcine pluripotent stem cells. FASEB J..

[69] Wang X., Pan C., Gong J., Liu X., Li H. (2016). Enhancing the enrichment of pharmacophore-based target prediction for the polypharmacological profiles of drugs. J. Chem. Inf. Model.

[70] Liu X., Ouyang S., Yu B., Liu Y., Huang K., Gong J. (2010). PharmMapper server: a web server for potential drug target identification using pharmacophore mapping approach. Nucleic Acids Res.

[71] Bu D., Luo H., Huo P., Wang Z., Zhang S., He Z. (2021). KOBAS-i: intelligent prioritization and exploratory visualization of biological functions for gene enrichment analysis. Nucleic Acids Res..

[72] Bugnon M., Röhrig U.F., Goullieux M., Perez M.A.S., Daina A., Michielin O. (2024). SwissDock 2024: major enhancements for small-molecule docking with Attracting Cavities and AutoDock Vina. Nucleic Acids Res..

[73] Röhrig U.F., Goullieux M., Bugnon M., Zoete V. (2023). Attracting cavities 2.0: improving the flexibility and robustness for small-molecule docking. J. Chem. Inf. Model..

[74] Budama-Kilinc Y., Cakir-Koc R., Kecel-Gunduz S., Kokcu Y., Bicak B., Mutlu H. (2018). Novel NAC-loaded poly(lactide-co-glycolide acid) nanoparticles for cataract treatment: preparation, characterization, evaluation of structure, cytotoxicity, and molecular docking studies. PeerJ.

[75] Abramson J., Adler J., Dunger J., Evans R., Green T., Pritzel A. (2024). Accurate structure prediction of biomolecular interactions with AlphaFold 3. Nature.

[76] Laskowski R.A., Swindells M.B. (2011). LigPlot+: multiple ligand-protein interaction diagrams for drug discovery. J. Chem. Inf. Model..

[77] McDonald I.K., Thornton J.M. (1994). Satisfying hydrogen bonding potential in proteins. J. Mol. Biol..

[78] Tierney L., Feigelson E.D., Babu G.J. (2012). Statistical Challenges in Modern Astronomy V.

