# Supplementary material for: Ginkgolide A enhances cardiomyocyte differentiation from pluripotent stem cells by targeting cytochrome c to attenuate intrinsic apoptosis
Source: J Biol Chem. 2026 Jun 6;302(7):113224. doi: 10.1016/j.jbc.2026.113224 (PMC13330671; doi:10.1016/j.jbc.2026.113224)
Supplement: Supplementary materials [file mmc1.docx]

Supplementary Table 1 Primers in this article

| Primer | Sequence |
| --- | --- |
| Hum-TNNT2-RP-LA | GGAGAGCAGAGACCATGTCTGACAGCCACAAACTTCTCTCTGCTA |
| Hum-TNNT2-Down-LA | TAGAAGAGGTGGTGGAAGAGTAC |
| Hum-TNNT2-Down-RA | GCCTTGTAAGTCATTGGTCTTAAAGGTACCTTTAGAAGGCACTGTTGTTGGA |
| Hum-TNNT2-RP-RA | CGTACTCTTCCACCACCTCTTCTAGTACCAGGCGGGGAGGC |
| hum-TNNT2-UP-LA | CGACGGCCAGTGCCAAGCTGACGCGTATTGACATAATTAAAGACTAGTACA |
| homo-TNNT2-gRNA-f | CACCGCACCTCTTCTATGTCAGACA |
| homo-TNNT2-gRNA-r | AAACTGTCTGACATAGAAGAGGTGC |
| q-homo-NANOG-F | CATGAGTGTGGATCCAGCTTG |
| q-homo-NANOG-R | CCTGAATAAGCAGATCCATGG |
| q-homo-OCT4-F | CCTTCGCAAGCCCTCATTTC |
| q-homo-OCT4-R | TAGCCAGGTCCGAGGATCAA |
| q-homo-TBXT-F | TATGAGCCTCGAATCCACATAGT |
| q-homo-TBXT-R | CCTCGTTCTGATAAGCAGTCAC |
| q-homo-MIXL1-F | GGCGTCAGAGTGGGAAATCC |
| q-homo-MIXL1-R | GGCAGGCAGTTCACATCTACC |
| q-homo-NKX2.5-F | CAAGTGTGCGTCTGCCTTTC |
| q-homo-NKX2.5-R | CGCGCACAGCTCTTTCTTT |
| q-homo-MESP1-F | CTGTTGGAGACCTGGATGCC |
| q-homo-MESP1-R | TCTGCCAAGGAACCACTTCG |
| q-homo-ISL1-F | GCGGAGTGTAATCAGTATTTGGA |
| q-homo-ISL1-R | GCATTTGATCCCGTACAACCT |
| q-homo-TBX5-F | AAGAGTTCCCTCCTCTCCCC |
| q-homo-TBX5-R | GTCTTGGCCCCGGGAATAAA |
| q-homo-TNNT2-F | TCCAGAAGACAGAGCGGAAA |
| q-homo-TNNT2-R | CTTCATTCAGGTGGTCAATGG |
| q-homo-GATA4-F | TCGTAGATATGTTTGACGACTTCT |
| q-homo-GATA4-R | AGTTGGCACAGGAGAGGC |


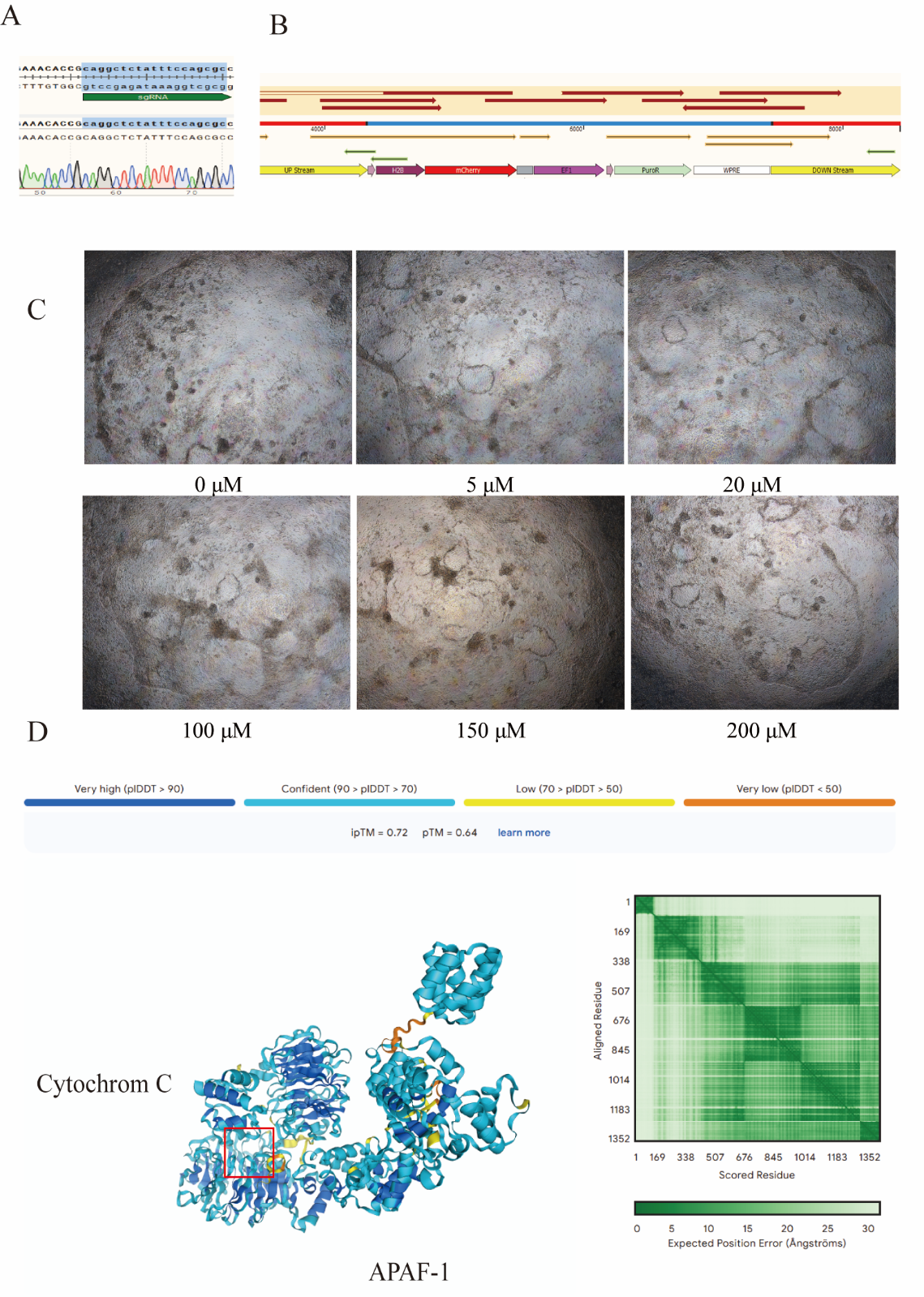


Fig S1 (A) Sanger sequencing chromatogram of the sgRNA insert ligated into the PX459 plasmid. (B) Sanger sequencing verification of the inserted fragment within the donor plasmid. (C) Phase-contrast images of hPSC-derived cardiomyocytes on induction date 8, treated with the indicated concentrations of Ginkgolide A. (D) Predicted complex structure of Cytochrome C and APAF-1 generated by AlphaFold 3, with the predicted protein-protein interaction interface highlighted in red.

Table S2 Pharmmapper_results of Ginkgolide A. (Data are available in the supplementary file “supplementary_table_2_pharmmapper_results.csv”.)

Table S3 DrugCLIP results of Ginkgolide A. (Data are available in the supplementary file “supplementary_table_3_DrugCLIP_results.csv”.)

Table S4 Enrichment results of predicted targets from GO and KEGG analyses. (Data are available in the supplementary file “supplementary_table_4_GO_KEGG_enrichment.csv”.)

Video 1 Rhythmic spontaneous contractions on Day 5 under 100 μM GA treatment. (See file “video1-GA100-D5.mp4”.)

Video 2 Rhythmic spontaneous contractions on Day 5 under 150 μM GA treatment. (See file “video2-GA150-D5.mp4”.)
